# Color-tunable up-conversion emission in Y_2_O_3_:Yb^3+^, Er^3+^ nanoparticles prepared by polymer complex solution method

**DOI:** 10.1186/1556-276X-8-131

**Published:** 2013-03-22

**Authors:** Vesna M Lojpur, Phillip S Ahrenkiel, Miroslav D Dramićanin

**Affiliations:** 1 Vinča Institute of Nuclear Sciences, University of Belgrade, P.O. Box 522, Belgrade, 11001, Serbia; 2 South Dakota School of Mines & Technology, Rapid City, SD, 57701, USA

**Keywords:** Luminescence, Up-conversion, Nanoparticles, Rare earth, Combustion

## Abstract

**Abstract:**

Powders of Y_2_O_3_ co-doped with Yb^3+^ and Er^3+^ composed of well-crystallized nanoparticles (30 to 50 nm in diameter) with no adsorbed ligand species on their surface are prepared by polymer complex solution method. These powders exhibit up-conversion emission upon 978-nm excitation with a color that can be tuned from green to red by changing the Yb^3+^/Er^3+^ concentration ratio. The mechanism underlying up-conversion color changes is presented along with material structural and optical properties.

**PACS:**

42.70.-a, 78.55.Hx, 78.60.-b

## Background

Up-conversion materials have the ability to convert lower energy near-infrared radiations into higher energy visible radiations. These materials have gained considerable attention because of their use in a wide range of important applications, from solid compact laser devices operating in the visible region and infrared quantum counter detectors to three-dimensional displays, temperature sensors, solar cells, anti-counterfeiting, and biological fluorescence labels and probes [1-6]. Further efforts in development of methods for preparation of up-conversion (UC) materials are therefore justified with aims of enhancing their UC efficiency and reducing production costs. In addition, methods for UC nanoparticle (UCNP) synthesis are of particular interest for use in two-photon bio-imaging, sensitive luminescent bio-labels, and GaAs-coated highly efficient light-emitting diodes [7].

Lanthanide-based UC materials and UCNPs are of special interest due to unique spectroscopic properties of rare-earth ions like sharp intra-4*f* electronic transitions and existence of abundant, long-living electronic excited states at various energies that facilitate electron promotion to high-energy states [8]. In principal, lanthanide-based UC materials and UCNPs consist of three components: a host matrix, a sensitizer, and an activator dopant. The choice of the host lattice determines the distance between the dopant ions, their relative spatial position, their coordination numbers, and the type of anions surrounding the dopant. The properties of the host lattice and its interaction with the dopant ions therefore have a strong influence on the UC process [9]. It has been shown that UC emission efficiency depends strongly on host phonon energy, where in low-phonon-energy hosts, multi-phonon relaxation processes are depressed and efficiency-enhanced [10]. Because of their excellent chemical stability, broad transparency range, and good thermal conductivity, rare-earth sesquioxides are well-suited host materials [11]. Their phonon energy (*ca.* 560 cm^−1^) is higher compared to the most UC-efficient fluoride materials (*ca.* 350 cm^−1^), but lower compared to other host types (phosphates, vanadates, molybdates, titanates, zirconates, silicates, etc.). In addition, easy doping can be achieved with RE ions because of similarity in ionic radius and charge. For sensitizer dopant, Yb^3+^ is the most common choice for excitation around 980 nm, where a variety of inexpensive optical sources exists. This ion has a simple energy level structure with two levels and a larger absorption cross section compared to other trivalent rare-earth ions. The energy separation of Yb^3+^^2^F_7/2_ ground state and ^2^F_5/2_ excited state match-up well the transitions of an activator dopant ion, which has easy charge transfer between its excited state and activator states. For visible emission, Er^3+^, Tm^3+^, Ho^3+^, and Pr^3+^ are commonly used as activator dopants [12-16]. UC emission of different colors can be obtained in a material with different activators and their combinations. Er^3+^-doped materials emit green and red light, Tm^3+^ blue, Ho^3+^ green, and Pr^3+^ red.

In recent times, a lot of effort is directed towards UC color tuning to obtain a material with characteristic emission usually by combining two or more activator ions [17] or by utilizing electron–electron and electron–phonon interactions in existing one-activator systems [18,19]. In this research we showed that color tuning from green to red can be achieved in Yb^3+^/Er^3+^ UCNP systems on account of changes of Yb^3+^ sensitizer concentration. For this purpose we prepared Y_2_O_3_ NPs, the most well-known rare-earth sesquioxide host, co-doped with different Yb^3+^/Er^3+^ ratios. Nanosized phosphors offer a number of potential advantages over traditional, micro-scale ones in optical properties, such as high-resolution images and high luminescence efficiency [20,21]. However, Vetrone et al. showed that CO_3_^2−^ and OH^−^ species are frequently adsorbed on the surface of sesquioxide nanoparticles [22]. Their high vibrational energies (about 1,500 and 3,350 cm^−1^ for CO_3_^2−^ and OH^−^, respectively) decrease the UC efficiency through multi-phonon relaxations. For this reason we applied polymer complex solution (PCS) synthesis [23] since we found earlier that the PCS method provides sesquioxides with low surface area and defects and no adsorbed species on the surface [24-26].

## Methods

### Sample fabrication

Polymer complex solution method is a modified combustion method where instead of classical fuel (urea, glycine, carbohydrazide) an organic water-soluble polymer (in our case polyethylene glycol (PEG)) is used. The utility of this polymeric approach comes from the coordination of metal cations on the polymer chains during gelation process, resulting in very low cation mobility. Polymer precursor works both as a chelating agent and as an organic fuel to provide combustion heat for the calcination process. In this way PCS provides mixing of constituting elements at the atomic level and allows homogeneous control of very small dopant concentration. The first step in the PCS method is preparation of an aqueous solution containing metal salts and PEG. In the second step, removal of the excess water forces polymer species into closer proximity, converting the system into a resin-like gel. Upon ignition, an oxide powder is obtained, while considerable resin mass is lost as the polymer matrix is burned away.

Using this procedure, three Y_2_O_3_ samples doped with 0.5 at.% of Er^3+^ and 1, 2.5, and 5 at.% of Yb^3+^ ions were synthesized. In brief, appropriate stoichiometric quantities of yttrium oxide (Y_2_O_3_), erbium oxide (Er_2_O_3_), and ytterbium oxide (Yb_2_O_3_) (all Alfa Aesar, 99.9%, Ward Hill, MA, USA) were mixed and dissolved in hot nitric acid. In the obtained solutions, PEG ($\bar{\mathrm{Mw}}$ = 200, Alfa Aesar) was added in 1:1 mass ratio. The formed metal-PEG solution was stirred at 80°C, resulting in a metal-PEG solid complex which was further fired at 800°C in air. The powders were additionally annealed at 800°C for 2 h in order to decompose the residual PEG and nitrite ions and to obtain pure crystal phase.

### Characterization methods

Crystal structures of samples are checked by X-ray diffraction (XRD) measurements. Measurements are performed on a Rigaku SmartLab system (Shibuya-ku, Japan) operating with Cu Kα_1,2_ radiation at 30 mA and 40 kV, in the 2*θ* range from 15° to 100° (using continuous scan of 0.7°/s). Transmission electron microscopy (TEM) is conducted using a JEOL-JEM 2100 instrument (Akishima-shi, Japan) equipped with LaB_6_ cathode and operated at 200 kV. The up-conversion luminescence emissions and decays are measured upon excitation with 978-nm radiation (OPO EKSPLA NT 342, 5.2-ns pulse, Vilnius, Lithuania) on a Horiba Jobin-Yvon Model FHR1000 spectrofluorometer system (Kyoto, Japan) equipped with an ICCD Jobin-Yvon 3771 detector. For measurements of up-conversion emission intensity dependence on excitation power, a continuous-wave laser is used (980-nm radiation).

## Results and discussion

**Figure 1:**
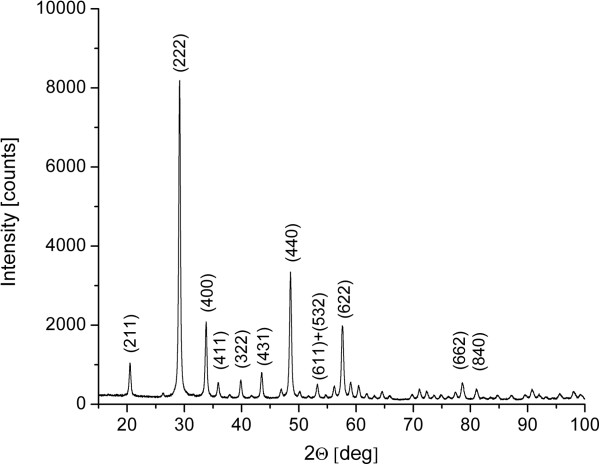
**XRD pattern of Y**_**1.97**_**Yb**_**0.02**_**Er**_**0.01**_**O**_**3 **_**UCNPs.** Diffraction peaks are indexed according to PDF card #87-2368 (cubic bixbyite Y_2_O_3_ crystal structure).

The representative XRD pattern for the Y_1.97_Yb_0.02_Er_0.01_O_3_-doped sample is shown in Figure 1. The XRD analysis confirms the presence of a cubic bixbyite Y_2_O_3_ crystal structure with space group *Ia-3* (no. 206), with diffraction peaks indexed according to the PDF card #87-2368. No other phases were detected and the small peak shifts in respect to pure Y_2_O_3_ are observed, indicating that Er^3+^ and Yb^3+^ ions have been effectively incorporated into the host lattice. An average crystallite size in the range of 21 nm is found by Halder-Wagner method analysis of all major diffraction peaks.

The presence of nitrate, water, and carbon species on nanoparticle surfaces is checked by Fourier transform infrared (FT-IR) spectroscopy. Only Y-O stretching vibrations of the host lattice at 560 cm^−1^ are noted (see Additional file 1: Figure S1 for the FT-IR spectrum of Y_1.97_Yb_0.02_Er_0.01_O_3_ sample). This is favorable for efficient emission since the high phonon energy of species adsorbed on the surface of nanoparticles may enhance significantly nonradiative de-excitation [13,22].

**Figure 2:**
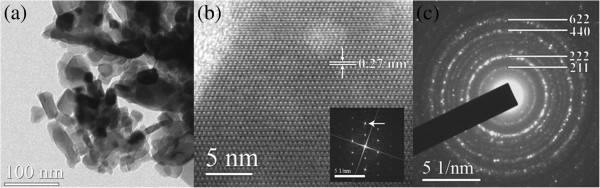
**TEM data from Y**_**1.97**_**Yb**_**0.02**_**Er**_**0.01**_**O**_**3 **_**sample.** (**a**) Bright-field image showing nanoparticle cluster. (**b**) [110] lattice image of a single particle. The 004 planes are indicated. Inset: FFT of image (indicated spot corresponds to 004 periodicity). (**c**) Selected-area diffraction pattern of nanoparticle cluster. Prominent planes are indexed.

The UCNPs are further investigated by transmission electron microscopy, and representative images are given in Figure 2. One can see highly agglomerated crystalline nanoparticles with irregular, polygonal-like shapes having a size in the range of 30 to 50 nm with boundary lines observed clearly in some regions (Figure 2a). Strong particle agglomeration is a main drawback of the PCS synthesis method. It is a consequence of an extremely high temperature gradient that occurs while firing metal-PEG complex. At that instance a large amount of high-pressure vapors is produced in the sample that strongly press particles onto each other. On the other hand, high-temperature gradients and pressure facilitate production of well-crystallized powder. An examination at higher magnifications (Figure 2b) reveals that grain boundaries are without any irregularities and that the surface of observed crystals is free of defects and without any amorphous layers. The spotty ring selected-area electron diffraction pattern (Figure 2c) confirms that Y_2_O_3_ powder is polycrystalline and is related to the fact that the constituent crystallites have a size of about 20 nm.

**Figure 3:**
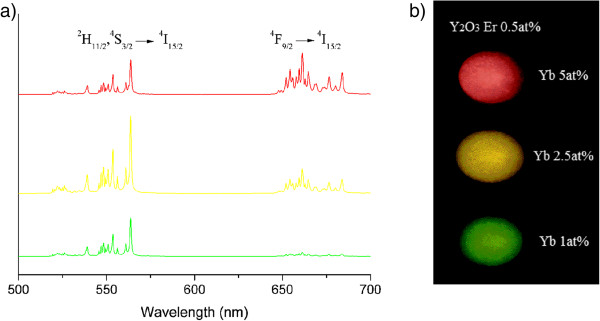
**UC spectra of NPs for all dopant compositions and photograph of pellets prepared from UCNPs.** (**a**) UC spectra of Y_1.97_Yb_0.02_Er_0.01_O_3_ (green line), Y_1.94_Yb_0.05_Er_0.01_O_3_ (yellow line), and Y_1.89_Yb_0.10_Er_0.01_O_3_ (red line) NPs. (**b**) Photograph of pellets prepared from UCNPs with different Yb^3+^ concentrations taken under 978-nm excitation.

**Figure 4:**
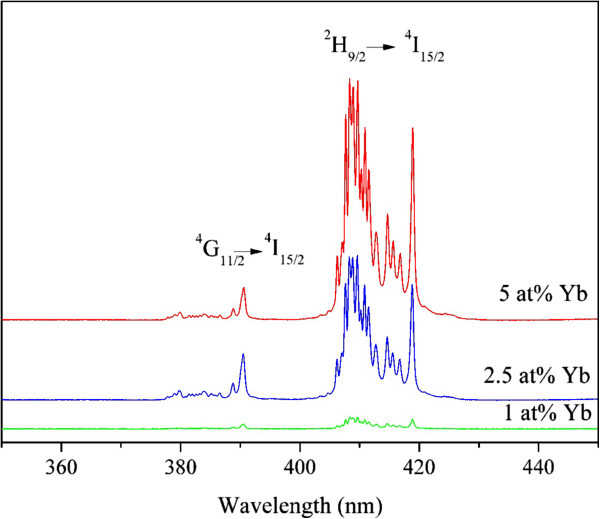
**UC spectra of NPs in UV-blue spectral region after excitation with 978-nm radiation.** Y_1.97_Yb_0.02_Er_0.01_O_3_ (green line), Y_1.94_Yb_0.05_Er_0.01_O_3_ (blue line), and Y_1.89_Yb_0.10_Er_0.01_O_3_ (red line).

The up-conversion luminescence spectra of NPs, for all Yb/Er dopant compositions, are measured upon excitation with 978-nm radiation. The main red and green emissions are shown in Figure 3a. They originate from Er^3+^*f*-*f* electronic transitions ^4^F_9/2_ → ^4^I_15/2_ (red emission) and (^2^H_11/2_, ^4^S_3/2_) → ^4^I_15/2_ (green emission) and are facilitated by the two-photon UC process. Weak emissions from higher photon order UC processes can be observed in the blue spectral (410 nm, ^2^H_9/2_ → ^4^I_15/2_ transition) and UV (390 nm, ^4^G_11/2_ → ^4^I_15/2_ transition) regions shown in Figure 4. These higher photon order emission diminishes in NPs with lower Yb^3+^ content (Y_1.97_Yb_0.02_Er_0.01_O_3_). The variation in Yb^3+^ concentration alters the red-to-green emission ratio (see Figure 3a), and consequently overall UC color of NPs is changed (see Figure 3b). The highest Yb^3+^ concentration of 5 at.% produces red color, and yellow is obtained with 2.5 at.% and green with 1 at.%.

**Figure 5:**
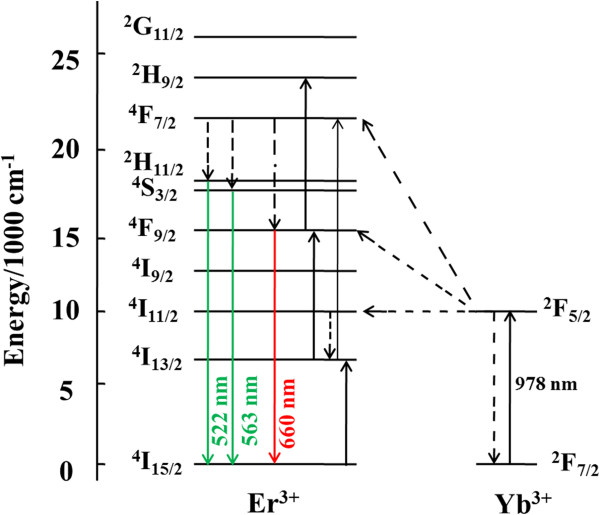
**Schematic energy level diagram showing the UC mechanism of Y**_**2**_**O**_**3**_**:Er**^**3+**^**, Yb**^**3+**^**.**

**Figure 6:**
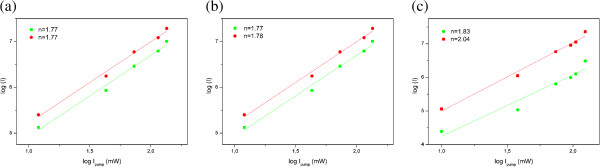
**Power dependence of UC emissions.** Dependence of the green (green line and symbols) and red (red line and symbols) UC emissions on excitation power for (**a**) Y_1.97_Yb_0.02_Er_0.01_O_3_, (**b**) Y_1.94_Yb_0.05_Er_0.01_O_3_, and (**c**) Y_1.89_Yb_0.10_Er_0.01_O_3_ NPs.

The energy level diagram of Yb^3+^ and Er^3+^ is shown in Figure 5 and illustrates the energy transfer from Yb^3+^ to Er^3+^ which generates up-conversion in a following manner: population of ^4^F_7/2_ level in Er^3+^ leads to an intermediate non-radiative relaxation to the ^2^H_11/2_ and ^4^S_3/2_ levels and further to two partially overlapped green emissions at 522 and 563 nm due to the radiative relaxations to the ^4^I_15/2_ level. Alternatively, the ^4^F_7/2_ level can partially non-radiatively relax to the ^4^F_9/2_ level from which red emission at 660 nm originates (^4^F_9/2_ → ^4^I_15/2_). Red emission could be intensified by another up-conversion path which occurs after non-radiate relaxation of the ^4^I_11/2_ to the ^4^I_13/2_ level, from where the additional population of the ^4^F_9/2_ level occurs through energy transfer. The population of the ^2^H_9/2_ level is realized by the excited state absorption from ^4^I_13/2_ and ^4^F_9/2_ levels. Blue up-conversion emission occurs by its radiative de-excitations to the ^4^I_15/2_ level. Power dependence of UC emissions, given in Figure 6, confirms that two-photon processes are responsible for green and red UC emissions. The observed slopes are similar for 1 and 2.5 at.% Yb^3+^-doped samples and slightly higher for 5 at.% Yb^3+^ doping.

Changes in red-to-green emission ratio with Yb^3+^ concentration increase in Y_2_O_3_:Er^3+^ bulk and NPs are discussed by Vetrone et al. [22]. They observed this phenomenon to be much more pronounced in NPs compared to bulk. They concluded that a cross-relaxation mechanism of ^4^F_7/2_ → ^4^F_9/2_ and ^4^F_9/2_ ← ^4^I_11/2_ is partly responsible for the red enhancement, but phonons of ligand species present on the NP surface enhance the probability of ^4^F_9/2_ level population from the ^4^I_13/2_ level. However, in the present case, no adsorbed species on the NPs are detected, as in other cases of NPs prepared with the PCS method. TEM images in Figure 2 and the Stark splitting of emission clearly evident in Figure 3a demonstrate the crystalline nature of NPs. Also, the values of UC emission decays, given in Table 1, are much larger compared to those from [22], indicating in this way the absence of a strong ligand influence on UC processes. Silver et al. [27] noticed that the Yb^3+^^2^F_5/2_ excited level may also receive electrons from higher energy levels of nearby Er^3+^ ions, back transferring energy from Er^3+^ to Yb^3+^ ions. When they compared spectra of Y_2_O_3_:Eu^3+^ with Yb^3+^, they noted that the up-conversion and down-conversion emissions lost intensity in the presence of Yb^3+^ and that was least apparent for the red ^4^F_9/2_ → ^4^I_15/2_ transition, even for a Yb^3+^/Er^3+^ ratio of 5:0.5. The decrease of ^4^F_9/2_ lifetime with Yb^3+^ concentration increase (Table 1) is a consequence of enlarged population of ^2^H_9/2_ by excited state absorption from the ^4^F_9/2_ level, which is evidenced through enhancement of blue emission (^2^H_9/2_ → ^4^I_15/2_) for larger Yb^3+^ content (see Figure 4).

**Table 1:** **Emission decay times for Y**_**2**_**O**_**3**_**:Yb**^**3+**^**, Er**^**3+ **^**nanoparticles upon 978-nm excitation**

	**Green emission lifetime (ms)**	**Red emission lifetime (ms)**
Y_1.97_Yb_0.02_Er_0.01_O_3_	0.36	0.71
Y_1.94_Yb_0.05_Er_0.01_O_3_	0.38	0.60
Y_1.89_Yb_0.10_Er_0.01_O_3_	0.34	0.35

## Conclusions

In conclusion, yttrium oxide powders doped with Er^3+^ ions and co-doped with different concentrations of Yb^3+^ ions are successfully prepared using polymer complex solution method. This simple and fast synthesis method provides powders consisting of well-crystallized nanoparticles (30 to 50 nm in diameter) with no adsorbed species on their surface. The powders exhibit up-conversion emission upon 978-nm excitation, with a color that can be tuned from green to red by changing the Yb^3+^/Er^3+^ concentration ratio. This effect can be achieved in nanostructured hosts where electron–phonon interaction is altered compared to the bulk material.

## Competing interests

The authors declare that they have no competing interests.

## Authors’ contributions

VL carried out the material synthesis. PA performed the TEM study. VL and MD carried out the X-ray diffraction and luminescence analysis. MD supervised the research activity. VL and MD wrote the manuscript. All authors discussed and commented on the manuscript. All authors approved the final manuscript.

## Supplementary Material

Additional file 1: Figure S1 FT-IR spectrum of Y_1.97_Yb_0.02_Er_0.01_O_3_. Click here for file
