# Supplementary figures and images for: Color-tunable up-conversion emission in Y2O3:Yb3+, Er3+ nanoparticles prepared by polymer complex solution method
Source: Nanoscale Res Lett. 2013 Mar 22;8(1):131. doi: 10.1186/1556-276X-8-131 (PMC3765681; doi:10.1186/1556-276X-8-131)

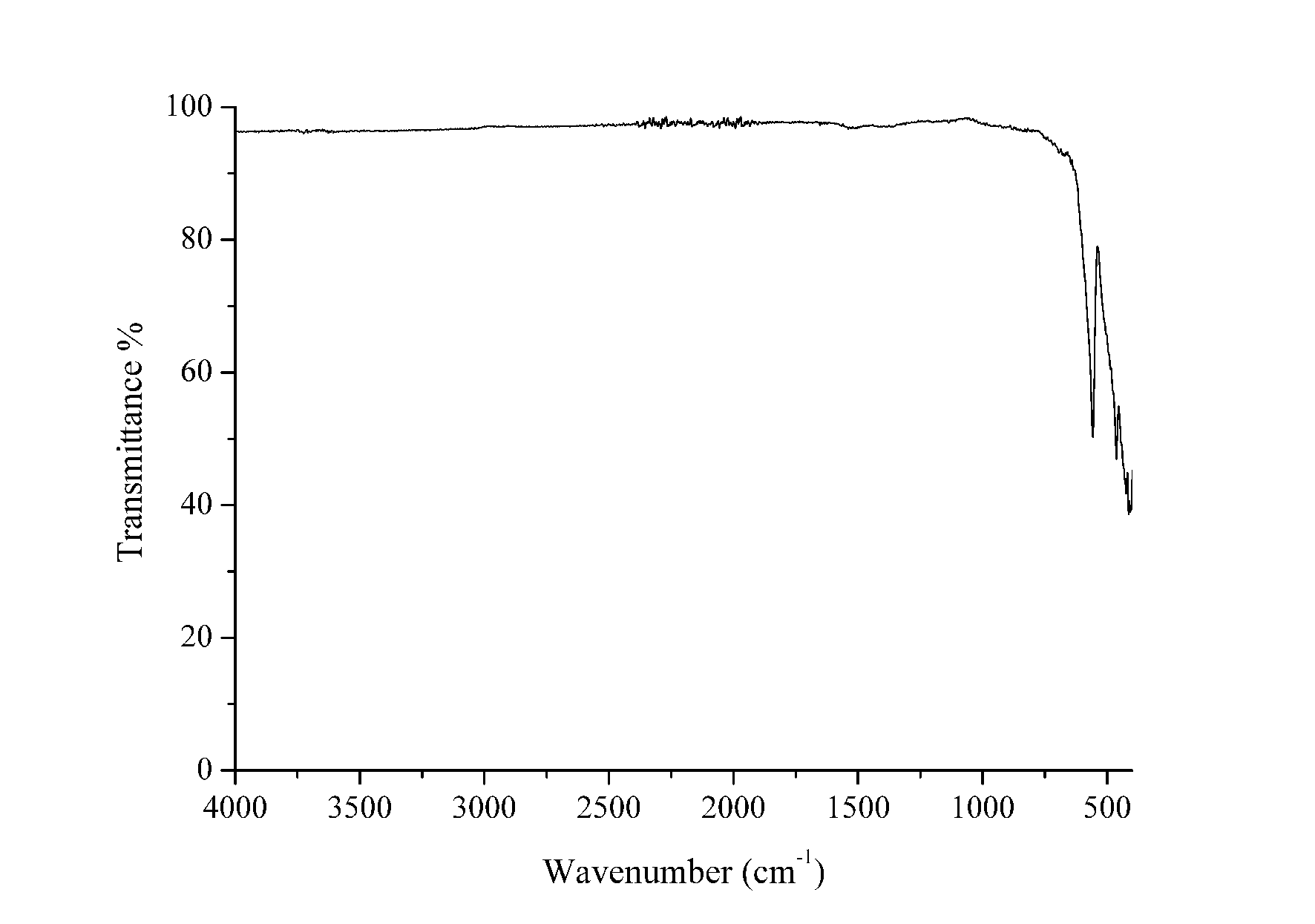

Supplement: Additional file 1: Figure S1 — FT-IR spectrum of Y1.97Yb0.02Er0.01O3. [file 1556-276X-8-131-S1.tiff]
